# Formation and Flocking Control Algorithms for Robot Networks with Double Integrator Dynamics and Time-Varying Formations

**DOI:** 10.3390/e25060834

**Published:** 2023-05-23

**Authors:** Carlos Montañez-Molina, Javier Pliego-Jiménez, Rigoberto Martínez-Clark

**Affiliations:** 1 Departamento de Electrónica y Telecomunicaciones, División de Física Aplicada, Centro de Investigación Científica y de Educación Superior de Ensenada, Ensenada 22860, Mexico; 2 Programa Investigadores por México, Consejo Nacional de Ciencia y Tecnología, Mexico City 03940, Mexico; 3 Facultad de Ingeniería, Administración y Ciencias Sociales, Universidad Autónoma de Baja California, Mexicali 21100, Mexico

**Keywords:** time-varying formation, flocking behavior, robot networks, hierarchical control, graph theory

## Abstract

In this work, we study the problem of designing control laws that achieve time-varying formation and flocking behaviors in robot networks where each agent or robot presents double integrator dynamics. To design the control laws, we adopt a hierarchical control approach. First, we introduce a virtual velocity, which is used as a virtual control input for the position subsystem (outer loop). The objective of the virtual velocity is to achieve collective behaviors. Then, we design a velocity tracking control law for the velocity subsystem (inner loop). An advantage of the proposed approach is that the robots do not require the velocity of their neighbors. Additionally, we address the case in which the second state of the system is not available for feedback. We include a set of simulation results to show the performance of the proposed control laws.

## 1. Introduction

Collective behaviors in nature are interesting phenomena that have attracted the attention of scientists from different disciplines. Interesting examples include flocks of birds and schools of fish, social networks, vehicular traffic, etc. In these examples, collective behaviors emerge due to local interactions between the members of the swarm. Such collective behaviors can be used to perform complex tasks; thus, the objective is to emulate them in groups of robots (in this work, the words agent and robot are used interchangeably), such as wheeled mobile robots, aerial and underwater vehicles, or robot manipulators. Controlling multiple agents presents some advantages, such as time reductions and energy savings when performing complex tasks. On the other hand, some of the applications that can be performed when replicating the collective behaviors in robot networks are search and rescue missions, object transportation, agricultural irrigation, and area mapping.

Examples of collective behaviors are consensus, rendezvous, synchronization, formation, and flocking. In this work, we only focus on formation and flocking behaviors in robot networks with double integrator dynamics. Currently, we can find a great variety of works that address these behaviors. A survey on formation control algorithms can be found in [1]. In this work, the authors classify the cases of study in formation control based on position, displacement, and distance measurements. In [2,3], the leader–follower approach is used to carry out the desired formation in multi-agent systems with double integrator dynamics. In [4,5], the leader–follower approach is also used, but these works consider time-varying formation problems. The problem of time-varying formation is more challenging than fixed formation since the geometric pattern of the formation changes over time. The problem of achieving time-varying formation with bounded control inputs is addressed in [5]. The authors present experimental results to validate the performance of the proposed controllers. A bearing-based formation control approach is presented in [6]. The control strategy allows one to track the time-varying leader velocities and to scale the geometry of the formation. A distributed control protocol that achieves time-varying formation for robot networks with linear dynamics is proposed in [7]. The authors introduce an adaptive coupling strength, which is updated according to the relative positions of the agents. A controller that allows one to accomplish time-varying formation robot networks with switching directed topologies is designed in [8]. A fault-tolerant time-varying formation control is proposed in [9]; to achieve the control goal, adaptive control theory is used. In [10], an affine formation maneuver algorithm that makes use of undirected graphs for the communication between agents is presented.

On the other hand, the flocking control problem mainly consists of causing all agents in the group to move to the same velocity while keeping a desired shape. In [11], the author proposes a control law that ensures the flocking behavior in robot networks with double integrator dynamics; it is important to point out that the controller also avoids collisions between agents and static obstacles. In [12], three control algorithms are proposed: the first two replicate the flocking behavior in free space and the third algorithm replicates the collective behavior in a space with obstacles; numerical results are shown to validate the three cases. A bounded control law based on potential fields that maintains a safe distance between the agents is presented in [13]. A flocking algorithm that allows the tracking of a virtual leader is studied in [14]; it is important to point out that in in this work, it is demonstrated that the flocking is achieved even when only some agents know the reference signals. The same problem is addressed in [15], where the authors consider the case wherein only some robots have access to the leader information. In addition, the authors employ adaptive control theory, which is the main difference with respect to [14]; moreover, the authors assume uncertainties and disturbances in the dynamic model of the robots. In [16], the problem of flocking is solved using an adaptive flocking control law, and the adaptive neural network estimates the uncertain nonlinear dynamics of the agents. The flocking problem with time delays is addressed in [17]. An optimal control approach is used to design flocking control in [18]; such an algorithm allows one to address obstacle avoidance and trajectory tracking problems. In [19], the authors propose a dynamic feedback position control law to address the problem. Such a control law does not require the velocity information of the agents to achieve the desired behavior. The flocking problem in discrete time is studied in [20]; the proposed algorithm is decentralized and allows the agents to converge to a formation based on flocking behavior, and simulations for the validation of the algorithm are shown. Unlike the mentioned works above, in [21,22,23], the authors design an observer to estimate the velocity, due to the fact that, in practice, it is not always possible to measure all the states of the system.

This work focuses on the design of control algorithms that achieve time-varying formation and flocking for robot networks where each node is modeled as a double integrator. The main contribution of this work is the decentralized dynamic hierarchical control law where, unlike the controllers reported in the literature, for the agent *i*, the proposed controller only requires the relative position of its neighbors and its own velocity. Additionally, the algorithm works for undirected and directed graphs. However, the graph must be connected. Furthermore, we consider the case wherein the velocity of the agents is not available from measurements, and, for the flocking case, only a few agents know the desired flocking velocity. To address the aforementioned problems, we propose Luenberger and distributed observers to estimate the unknown states and signals. Finally, we provide numerical simulations to show the effectiveness of the proposed control laws and observers. The remainder of the paper is organized as follows. Some preliminaries and the problem statement are given in Section 2. Formation and flocking control laws using the full state of the robots are developed in Section 3. The problem of time-varying formation and flocking without velocity measurements is studied in Section 4. Section 5 presents a distributed observer for the problem of flocking with partial information. Extensive simulations are reported in Section 6. Finally, the paper ends with some conclusions given in Section 7.

## 2. Preliminaries

### 2.1. Problem Statement

Consider a robot network with *N* identical elements whose dynamics are governed by double integrator dynamics

(1a) $$\begin{array}{cc} {\dot{p}}_{i} & =v_{i} \end{array}$$

(1b) $$\begin{array}{cc} {\dot{v}}_{i} & =u_{i} \end{array}$$

where the states of the *i*-th robot are the position $p_{i}\in {ℜ}^{n}$ and velocity $v_{i}\in {ℜ}^{n}$; $u_{i}\in {ℜ}^{n}$ is the control input and i∈N={1,2,…,N}. Next, we describe the collective behaviors under study.

**Formation**. The time-varying formation problem consists of steering all the robots of the team to a certain position to form a desired time-varying geometric shape or pattern while the velocity of each robot converges to the rate of change of the formation. The formation control objective can be defined as
  (2) $$\begin{array}{c} \lim_{t\to \infty }p_{ij}\left(t\right)-\delta_{ij}\left(t\right)=0\;\mathrm{and}\;\lim_{t\to \infty }v_{i}\left(t\right)={\dot{\delta }}_{i}\left(t\right)\;\forall i,j\in N \end{array}$$
  where $p_{ij}\left(t\right)=p_{i}\left(t\right)-p_{j}\left(t\right)\in {ℜ}^{n}$ is the error of position between the agent *i* and its neighbor denoted by *j*. $\delta_{ij}\left(t\right)=\delta_{i}\left(t\right)-\delta_{j}\left(t\right)\in {ℜ}^{n}$ describes the geometry of the formation and $\delta_{i}\left(t\right)\in {ℜ}^{n}$, $\delta_{j}\left(t\right)\in {ℜ}^{n}$ are time-varying offset vectors that are at least twice differentiable.
**Flocking**. In this collective behavior, all the robots follow a common reference velocity while maintaining a desired formation; thus, the flocking control objective is to achieve
  (3) $$\begin{array}{c} \lim_{t\to \infty }p_{ij}\left(t\right)-\delta_{ij}\left(t\right)=0\;\mathrm{and}\;\lim_{t\to \infty }v_{i}\left(t\right)=v^{d}\left(t\right)+{\dot{\delta }}_{i}\left(t\right)\;\forall i,j\in N \end{array}$$
  where $v^{d}\left(t\right)\in {ℜ}^{n}$ is the desired velocity for the team.

From the above definitions, the time-varying formation is a special case of flocking when $v^{d}\left(t\right)=0$.

### 2.2. Graph Theory

We consider that the communication topology is fixed and the communication channels can be either unidirectional or bidirectional. Such topologies can be modeled by undirected and directed graphs. A graph G=(N,E) consists of a set of nodes N (each node represents a robot in the network) and a set of edges E∈N×N. If the set of edges are unordered pairs of N, the graph is called undirected. For an undirected graph, the edge (i,j)∈E denotes that robots *i* and *j* can obtain information from each other. On the other hand, if the edges are ordered pairs of N, the graph is called a directed or digraph, and information only flows in one direction. The adjacency matrix $A=\left[a_{ij}\right]\in {ℜ}^{N\times N}$ for an undirected graph is defined as

(4) $$\begin{array}{c} a_{ij}=\left\{\begin{array}{cc} 1 & \mathrm{if}(i,j)\in E\\ 0 & \mathrm{otherwise} \end{array}\right. \end{array}$$

For a directed graph, we have

(5) $$\begin{array}{c} a_{ij}=\left\{\begin{array}{cc} 1 & j\in N_{i}\\ 0 & \mathrm{otherwise} \end{array}\right. \end{array}$$

where $N_{i}$ is the set of neighbors transmitting information to agent *i*. The Laplacian matrix $L=\left[\ell_{ij}\right]\in {ℜ}^{N\times N}$ is defined as

(6) $$\begin{array}{c} \ell_{ij}=\left\{\begin{array}{cc} \sum_{k=1}^{N}a_{ik} & i=j\\ -a_{ij} & i\neq j. \end{array}\right. \end{array}$$

For an undirected graph, the Laplacian matrix is symmetric and positive semidefinite. However, for a directed graph, *L* is not necessarily symmetric. By construction, the Laplacian matrix satisfies $L1_{N}=0$, meaning that the vector $1_{N}={\left[\begin{array}{ccc} 1 & \cdots & 1 \end{array}\right]}^{⊤}\in {ℜ}^{N}$ is an eigenvector of *L* and it is associated with the zero eigenvalue $\lambda_{1}=0$. For an undirected graph, all of the nonzero eigenvalues of *L* are real and positive, whereas, for a directed graph, the rest of the spectrum of *L* have positive real parts. If the eigenvalue $\lambda_{1}=0$ has algebraic multiplicity of one, then the graph is connected [24,25,26]. If there exists a sequence of edges (undirected path) that joins any pair of nodes, we say that the graph is connected [27]. A digraph is strongly connected if there is a directed path that connects every pair of nodes.

## 3. Formation and Flocking Controllers

In order to design the formation and flocking control laws, we exploit the cascade structure (chain of integrators) of the robot’s dynamics given in (1). To this end, we introduce the virtual input $\vartheta_{i}\in {ℜ}^{n}$ and rewrite (1) as follows:

(7a) $$\begin{array}{cc} {\dot{p}}_{i} & ={\tilde{v}}_{i}+\vartheta_{i} \end{array}$$

(7b) $$\begin{array}{cc} {\dot{\tilde{v}}}_{i} & =-{\dot{\vartheta }}_{i}+u_{i} \end{array}$$

where ${\tilde{v}}_{i}=v_{i}-\vartheta_{i}\in {ℜ}^{n}$ is the velocity error. The open-loop system (7) can be analyzed as an interconnected system with states $\left(p_{i},{\tilde{v}}_{i}\right)$ and inputs $\left(\vartheta_{i},u_{i}\right)$ coupled by the term ${\tilde{v}}_{i}$. Similar to the backstepping approach, the control problem is divided into two particular control problems: the first step consists of designing the control $\vartheta_{i}$ that achieves time-varying formation or flocking for the subsystem (7a), and as a second step, we design the control $u_{i}$ that steers the velocity error ${\tilde{v}}_{i}$ asymptotically to zero.

**Figure 1:**
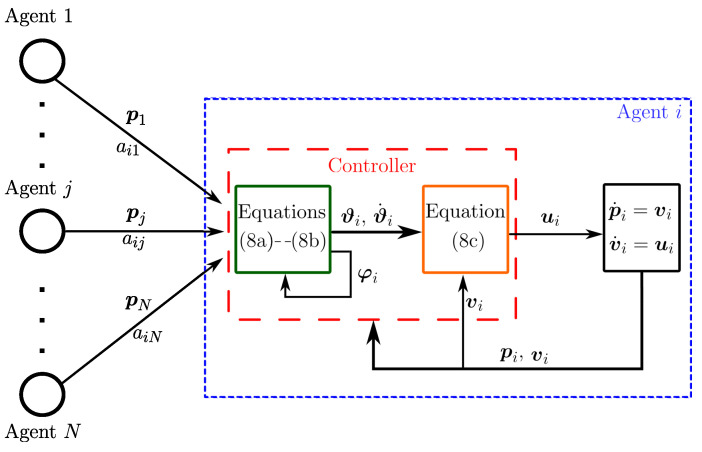
Block diagram of the proposed control for formation and flocking. In the green block, the external controller is included, while the orange block contains the internal controller; the values of the terms $a_{i1}$, $a_{ij}$ and $a_{iN}$ will change depending on the used graph.

The hierarchical control approach simplifies the control design; nevertheless, it imposes the restriction that the virtual input $\vartheta_{i}$ must be at least once differentiable; see (7b). Furthermore, it is desirable that the time derivative of control input $\vartheta_{i}$ for the *i*-th robot does not depend on the velocity of its neighbors. To fulfill the control objectives mentioned above, we propose the following dynamic control law:

(8a) $$\begin{array}{cc} \vartheta_{i} & =v^{d}\left(t\right)+{\dot{\delta }}_{i}\left(t\right)-k_{i}\varphi_{i} \end{array}$$

(8b) $$\begin{array}{cc} {\dot{\varphi }}_{i} & =-k_{i}\varphi_{i}+c\sum_{j=1}^{N}a_{ij}\left(p_{ij}-\delta_{ij}\left(t\right)-\varphi_{ij}\right) \end{array}$$

(8c) $$\begin{array}{cc} u_{i} & ={\dot{\vartheta }}_{i}-\gamma_{i}{\tilde{v}}_{i} \end{array}$$

where $v^{d}\left(t\right)\in {ℜ}^{n}$ is the desired flocking velocity that the robot network must follow, $\varphi_{i}\in {ℜ}^{n}$ is an additional state, $k_{i},\gamma_{i}\in ℜ$ are positive gains, c>0 is the coupling strength, $a_{ij}$ is the ij-th element of the adjacency matrix and $\varphi_{ij}=\varphi_{i}-\varphi_{j}$. In Figure 1, we present in a visual manner the proposed control law, so that readers have a better interpretation. Now, we are in a position to state the first result of this paper, which is summarized in the following proposition.

**Proposition** **1.** *Assume that the graph G is connected and consider the robot network *(1) *in a closed loop with *(8). *Then, the dynamic control law *(8) *guarantees*

*(i)*  *time-varying formation if $v^{d}\left(t\right)=0$;*
*(ii)*  *flocking behavior in the sense of* (3).

**Proof.**  To begin with stability analysis, we introduce the auxiliary state

(9) $$\begin{array}{c} r_{i}=p_{i}-p^{d}\left(t\right)-\delta_{i}\left(t\right)-\varphi_{i}\in {ℜ}^{n} \end{array}$$

where $p^{d}\left(t\right)=\int_{0}^{t}v^{d}\left(\tau \right)d\tau$. Then, it follows that ${\dot{r}}_{i}={\dot{p}}_{i}-v^{d}\left(t\right)-{\dot{\delta }}_{i}\left(t\right)-{\dot{\varphi }}_{i}$ and $r_{ij}=r_{i}-r_{j}=p_{ij}-\delta_{ij}\left(t\right)-\varphi_{ij}$. Substituting the control law (8) in (1) and taking into account (7), the closed-loop dynamics read

(10a) $$\begin{array}{cc} {\dot{r}}_{i} & =-c\sum_{j=1}^{N}a_{ij}r_{ij}+{\tilde{v}}_{i} \end{array}$$

(10b) $$\begin{array}{cc} {\dot{\varphi }}_{i} & =-k_{i}\varphi_{i}+c\sum_{j=1}^{N}r_{ij} \end{array}$$

(10c) $$\begin{array}{cc} {\dot{\tilde{v}}}_{i} & =-\gamma_{i}{\tilde{v}}_{i}. \end{array}$$

Using the properties of the Kronecker product, the closed-loop dynamics can be written in compact form as follows:

(11a) $$\begin{array}{cc} \dot{\varphi } & =-\left(K⊗I_{n}\right)\varphi +c\left(L⊗I_{n}\right)r \end{array}$$

(11b) $$\begin{array}{cc} \dot{r} & =-c\left(L⊗I_{n}\right)r+\tilde{v} \end{array}$$

(11c) $$\begin{array}{cc} \dot{\tilde{v}} & =-\left(\Gamma ⊗I_{n}\right)\tilde{v} \end{array}$$

where $\varphi ={\left[\varphi_{1}^{⊤}\;\cdots \;\varphi_{N}^{⊤}\right]}^{⊤}\in {ℜ}^{nN}$, $r={\left[r_{1}^{⊤}\;\cdots \;r_{N}^{⊤}\right]}^{⊤}\in {ℜ}^{nN}$, $\tilde{v}={\left[{\tilde{v}}_{1}^{⊤}\;\cdots \;{\tilde{v}}_{N}^{⊤}\right]}^{⊤}\in {ℜ}^{nN}$ are stacked vectors; $K=\mathrm{diag}\left\{k_{1},\cdots ,k_{N}\right\}\in {ℜ}^{nN\times nN}$, $\Gamma =\mathrm{diag}\left\{\gamma_{1},\cdots ,\gamma_{N}\right\}\in {ℜ}^{nN\times nN}$ are positive definite diagonal matrices; $I_{n}\in {ℜ}^{n\times n}$ is the identity matrix; and the symbol ⊗ denotes the Kronecker product. Using the fact $L1_{N}=0$, it can be shown that the closed-loop dynamics (11) have an equilibrium point at $\left(\varphi ,r,\tilde{v}\right)=\left(0,1_{N}⊗r^{★},0\right)$ for some $r^{★}\in {ℜ}^{n}$. To proceed with the stability analysis, we use the following change in coordinates [26]:

(12) $$\begin{array}{c} q=\left(Q⊗I_{n}\right)r\in {ℜ}^{n(N-1)} \end{array}$$

where the matrix $Q\in {ℜ}^{N-1\times N}$ is defined as

(13) $$\begin{array}{c} Q=\left[\begin{array}{ccccc} -1+(N-1)\vartheta & 1-\vartheta & -\vartheta & \cdots & -\vartheta \\ -1+(N-1)\vartheta & -\vartheta & 1-\vartheta & \ddots & \vdots \\ \vdots & \vdots & \ddots & \ddots & -\vartheta \\ -1+(N-1)\vartheta & -\vartheta & \cdots & -\vartheta & 1-\vartheta \end{array}\right] \end{array}$$

with

$$\vartheta =\frac{\left(N-\sqrt{N}\right)}{N(N-1)}.$$

By construction, the matrix *Q* satisfies [28]

(14) $$\begin{array}{c} Q1_{N}=0,\;QQ^{⊤}=I_{N-1},\;Q^{⊤}Q=I_{N}-\frac{1}{N}1_{N}1_{N}^{⊤}. \end{array}$$

Using these properties, it can be shown that

(15) $$\begin{array}{c} q=\left(Q⊗I_{n}\right)r=0\Rightarrow r=1_{N}⊗r^{★} \end{array}$$

Finally, by taking into account (12), (14) and

$$r=\left(Q^{⊤}⊗I_{n}\right)q+\frac{1}{N}\left(1_{N}1_{N}^{⊤}⊗I_{n}\right)r,$$

the closed-loop dynamics (11) become

(16) $$\begin{array}{c} \dot{\xi }=A_{\xi }\xi \end{array}$$

where $\xi ={\left[\begin{array}{ccc} \varphi^{⊤} & q^{⊤} & \tilde{v} \end{array}\right]}^{⊤}\in {ℜ}^{3nN-n}$ is the extended state and

$$A_{\xi }=\left[\begin{array}{ccc} -K⊗I_{n} & c(LQ^{⊤}⊗I_{n}) & O_{nN}\\ O_{n(N-1)\times nN} & -c(L_{r}⊗I_{n}) & Q⊗I_{n}\\ O_{nN} & O_{nN\times n(N-1)} & -\Gamma ⊗I_{n} \end{array}\right]$$

where $L_{r}=QLQ^{⊤}\in {ℜ}^{N-1\times N-1}$ is the reduced Laplacian matrix; $O_{nN\times nN}\in {ℜ}^{nN\times nN}$ and $O_{n(N-1)\times nN}\in {ℜ}^{nN\times nN}$ denote zero matrices. Since G is a connected graph by assumption, the real parts of the eigenvalues of $L_{r}$ are positive [26]. Moreover, the eigenvalues of $L_{r}$ are the same as the Laplacian matrix *L* except for $\lambda_{1}=0$. This implies that the matrix $-cL_{r}$ is Hurwitz. Since each matrix on the diagonal of $A_{\xi }$ is a Hurwitz matrix and $A_{\xi }$ is a block upper triangular matrix, it follows that $A_{\xi }$ is also Hurwitz. Therefore, the origin of (16) (ξ=0) is a globally exponentially stable equilibrium point.

From Equation (15), the exponential convergence of q(t) to zero implies $r\left(t\right)\to 1_{N}⊗r^{★}$ as t→∞ and hence $r_{i}\left(t\right)\to r^{★}$, which in turn implies $r_{ij}\left(t\right)\to 0$. Combining the previous result with the exponential convergence of φ(t) to zero yields

$$\lim_{t\to \infty }p_{ij}\left(t\right)-\delta_{ij}\left(t\right)=r_{ij}\left(t\right)+\varphi_{ij}\left(t\right)=0,\;i,j\in N.$$

This implies that the robot network achieves the desired geometry formation. The last part of the proof consists of showing that the robots’ velocities satisfy $\lim_{t\to \infty }v_{i}\left(t\right)={\dot{\delta }}_{i}\left(t\right)$ (∀i∈N) for time-varying formation and $\lim_{t\to \infty }v_{i}\left(t\right)=v^{d}\left(t\right)+{\dot{\delta }}_{i}\left(t\right)$ for the flocking behavior. This can be done by noticing that $\lim_{t\to \infty }{\tilde{v}}_{i}\left(t\right)=v_{i}\left(t\right)-\vartheta_{i}\left(t\right)=0$, and from (8a) one has $\lim_{t\to \infty }v_{i}\left(t\right)=\vartheta_{i}\left(t\right)=v^{d}\left(t\right)+{\dot{\delta }}_{i}\left(t\right)-k_{i}\varphi_{i}\left(t\right)$. Since $\varphi_{i}\left(t\right)\to 0$ as t→∞, it follows that

$$\lim_{t\to \infty }v_{i}\left(t\right)=v^{d}\left(t\right)+{\dot{\delta }}_{i}\left(t\right)$$

meaning that the flocking behavior is achieved. Finally, for time-varying formation ($v^{d}\left(t\right)=0$), we have $\lim_{t\to \infty }v_{i}\left(t\right)-\vartheta_{i}\left(t\right)=v_{i}\left(t\right)-{\dot{\delta }}_{i}\left(t\right)+k_{i}\varphi_{i}\left(t\right)=0$ and hence

$$\lim_{t\to \infty }v_{i}\left(t\right)={\dot{\delta }}_{i}\left(t\right)$$

which implies that the time-varying formation is achieved. □

## 4. Time-Varying Formation and Flocking Controllers without Velocity Measurements

**Figure 2:**
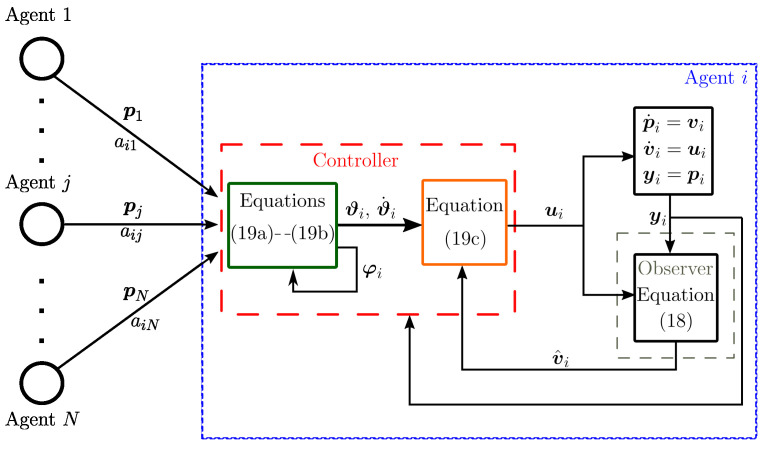
Block diagram of the proposed control for formation and flocking using the observer.

Motivated by the fact that many commercial robots are only equipped with position sensors, in this section, we combine the dynamic control law (8) with a linear observer that estimates the second state for the robot *i*. Consider the robot network

(17a) $$\begin{array}{cc} {\dot{p}}_{i} & =v_{i} \end{array}$$

(17b) $$\begin{array}{cc} {\dot{v}}_{i} & =u_{i} \end{array}$$

(17c) $$\begin{array}{cc} y_{i} & =p_{i},\;\forall i\in N \end{array}$$

where $y_{i}\in {ℜ}^{n}$ is the output of the system. To address the lack of velocity measurements, we propose the observer

(18a) $$\begin{array}{cc} {\dot{\hat{p}}}_{i} & ={\hat{v}}_{i}+\Xi_{1i}{\tilde{y}}_{i} \end{array}$$

(18b) $$\begin{array}{cc} {\dot{\hat{v}}}_{i} & =u_{i}+\Xi_{2i}{\tilde{y}}_{i} \end{array}$$

(18c) $$\begin{array}{cc} {\tilde{y}}_{i} & =p_{i}-{\hat{p}}_{i} \end{array}$$

where ${\hat{p}}_{i}\in {ℜ}^{n}$ and ${\hat{v}}_{i}\in {ℜ}^{n}$ denote, respectively, the estimated position and estimated velocity; ${\tilde{y}}_{i}\in {ℜ}^{2}$ is the output error and $\Xi_{1i}\in {ℜ}^{n\times n}$, $\Xi_{2i}\in {ℜ}^{n\times n}$ are the observer gains. In Figure 2, we show a block diagram that depicts the controller when including the Luenberger observer.

**Proposition** **2.** *Consider the robot network *(17) *and the observer* (18) *in a closed loop with the dynamic controller*

(19a) $$\begin{array}{cc} \vartheta_{i} & =v^{d}\left(t\right)+{\dot{\delta }}_{i}\left(t\right)-k_{i}\varphi_{i} \end{array}$$

(19b) $$\begin{array}{cc} {\dot{\varphi }}_{i} & =-k_{i}\varphi_{i}+c\sum_{j=1}^{N}a_{ij}\left(p_{ij}-\delta_{ij}\left(t\right)-\varphi_{ij}\right) \end{array}$$

(19c) $$\begin{array}{cc} u_{i} & ={\dot{\vartheta }}_{i}-\gamma_{i}\left({\hat{v}}_{i}-\vartheta_{i}\right) \end{array}$$

*where $v^{d}\left(t\right)\in {ℜ}^{n}$ is the desired flocking velocity, $k_{i},\gamma_{i}\in ℜ$ are positive gains, c>0 is the coupling strength, and ${\hat{v}}_{i}\in {ℜ}^{n}$ is the estimated velocity. Assume that the communication graph G is connected and the observer gain $\Xi_{i}={\left[\begin{array}{cc} \Xi_{1i} & \Xi_{2i} \end{array}\right]}^{⊤}\in {ℜ}^{2n\times n}$ is chosen such that the matrix $A_{i}-\Xi_{i}C_{i}$ is Hurwitz where*

$$\begin{array}{c} A_{i}=\left[\begin{array}{cc} O_{n} & I_{n}\\ O_{n} & O_{n} \end{array}\right],\;C_{i}=\left[\begin{array}{cc} I_{n} & O_{n} \end{array}\right]. \end{array}$$

*Then, the controller* (19) *in combination with the observer* (18) *guarantees*

*(i)*  *time-varying formation if $v^{d}\left(t\right)=0$;*
*(ii)*  *flocking as defined in *(3);
*(iii)*  *${\hat{v}}_{i}\left(t\right)\to v_{i}\left(t\right)$ as t→∞.*

**Proof.**  First, we define the observer error ${\tilde{x}}_{i}={\left[\begin{array}{cc} {\left(p_{i}-{\hat{p}}_{i}\right)}^{⊤} & {\left(v_{i}-{\hat{v}}_{i}\right)}^{⊤} \end{array}\right]}^{⊤}\in {ℜ}^{2n}$. By taking into account (17) and (18), the time derivative of ${\tilde{x}}_{i}$ is given by

(20) $$\begin{array}{c} {\dot{\tilde{x}}}_{i}=A_{oi}{\tilde{x}}_{i} \end{array}$$

where $A_{oi}=A_{i}-\Xi_{i}C_{i}\in {ℜ}^{2n\times 2n}$. By taking into account the observer error dynamics (20), the robot network (17) and the control law (19), the overall closed-loop dynamics are given by

(21a) $$\begin{array}{cc} {\dot{\varphi }}_{i} & =-k_{i}\varphi_{i}+c\sum_{j=1}^{N}a_{ij}r_{ij} \end{array}$$

(21b) $$\begin{array}{cc} {\dot{r}}_{i} & =-c\sum_{j=1}^{N}a_{ij}r_{ij}+{\tilde{v}}_{i} \end{array}$$

(21c) $$\begin{array}{cc} {\dot{\tilde{v}}}_{i} & =-\gamma_{i}{\tilde{v}}_{i}+E_{i}{\tilde{x}}_{i} \end{array}$$

(21d) $$\begin{array}{cc} {\dot{\tilde{x}}}_{i} & =A_{oi}{\tilde{x}}_{i} \end{array}$$

where $E_{i}=\left[\begin{array}{cc} O_{n} & \gamma_{i}I_{n} \end{array}\right]\in {ℜ}^{n\times 2n}$ and $r_{i}$ is defined in (9). Notice that the addition of the observer error dynamics does not destroy the cascade structure of the closed-loop dynamics. Therefore, we can follow the steps of the proof of Proposition 1 to obtain

(22) $$\begin{array}{c} \dot{\eta }=A_{\eta }\eta \end{array}$$

where $\eta ={\left[\begin{array}{cccc} \varphi^{⊤} & q^{⊤} & {\tilde{v}}^{⊤} & {\tilde{x}}^{⊤} \end{array}\right]}^{⊤}\in {ℜ}^{5nN-n}$ is the extended state vector, $q\in {ℜ}^{n(N-1)}$ is defined in (12), $\tilde{x}={\left[{\tilde{x}}_{1}^{⊤}\;\cdots \;{\tilde{x}}_{N}^{⊤}\right]}^{⊤}\in {ℜ}^{2nN}$, and $A_{\eta }$ is a block upper triangular matrix given by

(23) $$\begin{array}{c} A_{\eta }=\left[\begin{array}{cccc} -K⊗I_{n} & c(LQ^{⊤}⊗I_{n}) & O_{nN} & O_{nN\times 2nN}\\ O_{n(N-1)\times nN} & -c(L_{r}⊗I_{n}) & Q⊗I_{n} & O_{n(N-1)\times 2nN}\\ O_{nN} & O_{nN\times n(N-1)} & -\Gamma ⊗I_{n} & -E\\ O_{nN\times 2nN} & O_{2nN\times n(N-1)} & O_{2nN\times nN} & A_{o} \end{array}\right] \end{array}$$

with $A_{o}=\mathrm{blockdiag}\left\{A_{o1},\cdots ,A_{oN}\right\}\in {ℜ}^{2nN\times 2nN}$ and

$$E=\left[\begin{array}{ccc} E_{1} & \cdots & O_{n\times 2n}\\ \vdots & \ddots & \vdots \\ O_{n\times 2n} & \cdots & E_{N} \end{array}\right].$$

Since the observer gain $\Xi_{i}$ guarantees that $A_{oi}=A_{i}-\Xi_{i}C_{i}$ is Hurwitz, each matrix on the diagonal of $A_{\eta }$ is Hurwitz; thus, the equilibrium point η=0 is exponentially stable. The previous result directly implies that

$$\lim_{t\to \infty }{\hat{v}}_{i}\left(t\right)=v_{i}\left(t\right),$$

and hence item (iii) is proven. Due to the fact that φ(t), q(t) and $\tilde{v}\left(t\right)$ converge exponentially to zero, we can follow the steps of the proof of Proposition 1 to prove items (i) and (ii). □

## 5. Flocking Control with Partial Information

In order to design the controllers (8) and (19), it is assumed that all the robots have access to the desired flocking velocity $v^{d}\left(t\right)$. In this section, we study the scenario in which only a portion of the robots have access to the flocking velocity. Let $N^{d}\subset N$ be the subset that contains all the robots that have access to $v^{d}\left(t\right)$. Since at least one robot knows $v^{d}\left(t\right)$, it is possible to estimate the flocking velocity for the robot $i\notin N^{d}$ by using information about its neighbors.

**Proposition** **3.** *Assume that G is undirected (directed) and connected (strongly connected). Moreover, assume that the desired flocking velocity $v^{d}\left(t\right)\in {ℜ}^{n}$ is a bounded continuous function and satisfies that ${\dot{v}}^{d}\left(t\right)\to 0$ as t→∞. Then, the distributed observer*

(24) $$\begin{array}{c} {\dot{\hat{v}}}_{i}^{d}=-\mu \left(\sum_{j=1}^{N}a_{ij}{\hat{v}}_{ij}^{d}+d_{i}\left({\hat{v}}_{i}^{d}-v^{d}\left(t\right)\right)\right) \end{array}$$

*where μ>0 is the observer gain, ${\hat{v}}_{i}^{d}\left(t\right)\in {ℜ}^{n}$ is an estimate of $v^{d}\left(t\right)$, ${\hat{v}}_{ij}^{d}={\hat{v}}_{i}^{d}-{\hat{v}}_{j}^{d}$ and*

(25) $$\begin{array}{c} d_{i}=\left\{\begin{array}{cc} 1, & \mathrm{if}\;i\in N^{d}\\ 0 & otherwise \end{array}\right. \end{array}$$

*guarantees that ${\hat{v}}_{i}^{d}\left(t\right)\to v^{d}\left(t\right)$ as t→∞.*

**Proof.**  The flocking estimation error is defined as ${\bar{v}}_{i}^{d}≜{\hat{v}}_{i}^{d}-v^{d}\left(t\right)$ and, from (24), its time derivative is given by

(26) $$\begin{array}{c} {\dot{\bar{v}}}_{i}^{d}=-\mu \left(\sum_{j=1}^{N}a_{ij}{\bar{v}}_{ij}^{d}+d_{i}{\bar{v}}_{i}^{d}\right)-{\dot{v}}^{d}\left(t\right) \end{array}$$

where we use the fact that ${\hat{v}}_{i}^{d}-{\hat{v}}_{j}^{d}={\bar{v}}_{i}^{d}-{\bar{v}}_{j}^{d}$. Using again the Kronecker product, (26) becomes

(27) $$\begin{array}{cc} {\dot{\bar{v}}}^{d} & =-\mu \left(L⊗I_{n}\right){\bar{v}}^{d}-\mu \left(D⊗I_{n}\right){\bar{v}}^{d}-1_{N}⊗{\dot{v}}^{d}\left(t\right)\\ & =-\mu \left(F⊗I_{n}\right){\bar{v}}^{d}-1_{N}⊗{\dot{v}}^{d}\left(t\right) \end{array}$$

where ${\bar{v}}^{d}={\left[{\left({\bar{v}}_{1}^{d}\right)}^{⊤}\;\cdots \;{\left({\bar{v}}_{N}^{d}\right)}^{⊤}\right]}^{⊤}\in {ℜ}^{nN}$, $D=\mathrm{diag}\left\{d_{1},\ldots ,d_{N}\right\}\in {ℜ}^{N\times N}$ and $F=L+D\in {ℜ}^{N\times N}$. Since G is undirected (directed) and connected (strongly connected) by assumption, the eigenvalues of *F* are strictly positive (see [29] for further details). This implies that the matrix $-\mu (F⊗I_{n})$ is Hurwitz. Thus, if ${\dot{v}}^{d}\left(t\right)=0$, the system (27) has an exponentially stable equilibrium point at the origin. The differential Equation (27) can be analyzed as a stable linear system with a continuous bounded input ${\dot{v}}^{d}\left(t\right)$ that converges asymptotically to zero [30]. Therefore, we can conclude that

$$\lim_{t\to \infty }{\bar{v}}^{d}\left(t\right)=0.$$

Therefore, ${\hat{v}}_{i}^{d}\left(t\right)\to v^{d}\left(t\right)$ as t→∞ for all i∈N. □

The results of Propositions 2 and 3 can be combined to solve the problem of flocking without velocity measurements and with partial information.

**Proposition** **4.** *Consider the robot network* (1) *in a closed loop with the flocking control law*

(28a) $$\begin{array}{cc} \vartheta_{i} & ={\hat{v}}_{i}^{d}\left(t\right)+{\dot{\delta }}_{i}\left(t\right)-k_{i}\varphi_{i} \end{array}$$

(28b) $$\begin{array}{cc} {\dot{\varphi }}_{i} & =-k_{i}\varphi_{i}+c\sum_{j=1}^{N}a_{ij}\left(p_{ij}-\delta_{ij}\left(t\right)-\varphi_{ij}\right),\;\varphi_{i}\left(0\right)=\varphi_{i0}\in {ℜ}^{n} \end{array}$$

(28c) $$\begin{array}{cc} u_{i} & ={\dot{\vartheta }}_{i}-\gamma_{i}\left({\hat{v}}_{i}-v_{i}^{a}\right) \end{array}$$

*where ${\hat{v}}_{i}\in {ℜ}^{n}$ and ${\hat{v}}_{i}^{d}\in {ℜ}^{n}$ are obtained from the observers* (18) *and* (24), *respectively. Assume that G is undirected (directed) and connected (strongly connected). Moreover, assume that the desired flocking velocity $v^{d}\left(t\right)\in {ℜ}^{n}$ is a bounded continuous function and satisfies that ${\dot{v}}^{d}\left(t\right)\to 0$ as t→∞. Then, the controller* (28) *in combination with the observers* (18) *and* (24) *achieves flocking in the sense of* (3).

**Proof.**  By taking into account (17), (7), (18), (24) and (28), the closed-loop dynamics read

(29a) $$\begin{array}{cc} {\dot{\varphi }}_{i} & =-k_{i}\varphi_{i}+c\sum_{j=1}^{N}a_{ij}r_{ij} \end{array}$$

(29b) $$\begin{array}{cc} {\dot{r}}_{i} & =-c\sum_{j=1}^{N}a_{ij}r_{ij}+{\tilde{v}}_{i}+{\bar{v}}_{i}^{d} \end{array}$$

(29c) $$\begin{array}{cc} {\dot{\tilde{v}}}_{i} & =-\gamma_{i}{\tilde{v}}_{i}+E_{i}{\tilde{x}}_{i} \end{array}$$

(29d) $$\begin{array}{cc} {\dot{\tilde{x}}}_{i} & =A_{oi}{\tilde{x}}_{i} \end{array}$$

(29e) $$\begin{array}{cc} {\dot{\bar{v}}}_{i}^{d} & =-\mu \left(\sum_{j=1}^{N}\left(a_{ij}+d_{i}\right){\bar{v}}_{ij}^{d}\right)-{\dot{v}}^{d}\left(t\right). \end{array}$$

Using (12) and (14), the closed-loop dynamics can be written as

(30) $$\begin{array}{c} \dot{\sigma }=A_{\sigma }\sigma +B_{\sigma }{\dot{v}}^{d}\left(t\right) \end{array}$$

where $\sigma ={\left[\begin{array}{ccccc} \varphi^{⊤} & q^{⊤} & {\tilde{v}}^{⊤} & {\tilde{x}}^{⊤} & {\left({\bar{v}}^{d}\right)}^{⊤} \end{array}\right]}^{⊤}\in {ℜ}^{6nN-n}$ and

$$\begin{array}{c} A_{\sigma }=\left[\begin{array}{ccccc} -K⊗I_{n} & c(LQ^{⊤}⊗I_{n}) & O_{nN} & O_{nN\times 2nN} & O_{nN}\\ O_{n(N-1)\times nN} & -c(L_{r}⊗I_{n}) & Q⊗I_{n} & O_{n(N-1)\times 2nN} & Q⊗I_{n}\\ O_{nN} & O_{nN} & -\Gamma ⊗I_{n} & E & O_{nN}\\ O_{2nN\times nN} & O_{2nN\times n(N-1)} & O_{2nN\times nN} & A_{o} & O_{2nN\times nN}\\ O_{nN} & O_{nN\times n(N-1)} & O_{nN} & O_{nN\times 2nN} & -\mu (F⊗I_{n}) \end{array}\right] \end{array}$$

and

$$\begin{array}{c} B_{\sigma }={\left[\begin{array}{ccccc} O_{nN} & O_{nN\times n(N-1)} & O_{nN} & O_{nN\times 2nN} & I_{n} \end{array}\right]}^{⊤}. \end{array}$$

It can be verified that $A_{\sigma }$ is Hurwitz and hence σ=0 is a globally exponentially stable equilibrium point if ${\dot{v}}^{d}\left(t\right)=0$. Therefore, the closed-loop dynamics (30) are a stable linear system with input ${\dot{v}}^{d}\left(t\right)$ that converges to zero. Thus, we conclude that σ(t)→0 as t→∞. This in turn implies that ${\hat{v}}_{i}\left(t\right)\to v_{i}$ and ${\hat{v}}_{i}^{d}\left(t\right)\to v^{d}\left(t\right)$ as t→∞. Following the steps of Proposition 1, it can be shown that $p_{ij}\left(t\right)-\delta_{ij}\left(t\right)\to 0$ and $v_{i}\left(t\right)\to v^{d}\left(t\right)+{\dot{\delta }}_{i}\left(t\right)$ as t→∞. □

## 6. Numerical Results

In this section, we present numerical results to show the performance of the proposed control laws and observers. To carry out the simulation, we consider a robot network with six agents represented by (1). The simulations are carried out on Matlab software. In each simulation, it is assumed that only the position of each robot is available from sensor measurements; thus, the observer (18) is used in combination with the formation and flocking controllers.

### 6.1. Time-Varying Formation Control

First, we validate the performance of the time-varying formation that is obtained from (19) by setting $v^{d}\left(t\right)=0$. All the robots start at rest, i.e., $v_{i}\left(0\right)=0$, and the initial position of each agent is chosen as

$$\begin{array}{cc} p_{1}\left(0\right) & ={\left[-0.36\;\;0.9\right]}^{⊤},\;\;p_{2}\left(0\right)={\left[1.8\;\;1.8\right]}^{⊤},\;\;p_{3}\left(0\right)={\left[-1.8\;\;2.7\right]}^{⊤},\\ p_{4}\left(0\right) & ={\left[2.7\;\;2.7\right]}^{⊤},\;\;p_{5}\left(0\right)={\left[-2.7\;\;-1.8\right]}^{⊤},\;\;p_{6}\left(0\right)={\left[2.7\;\;-0.9\right]}^{⊤}. \end{array}$$

The initial conditions for the Luenberger observer states were set as ${\hat{p}}_{i}\left(0\right)={\hat{v}}_{i}\left(0\right)=0$ for all i∈N. On the other hand, the initial condition for the auxiliary state $\varphi_{i}$ was set as $\varphi_{i}\left(0\right)=0$ for all i∈N. The control and observer gains are, respectively, chosen as $K=\Gamma =5I\in {ℜ}^{12\times 12}$ and $\Xi_{i}={\left[\begin{array}{cc} 6I & 5I \end{array}\right]}^{⊤}\in {ℜ}^{4\times 2}$, while the coupling strength is set as c=2.

To achieve the desired time-varying formation, we use the following

$$\delta \left(t\right)=\delta_{0}+\delta_{1}\tanh\left(at-6\right)$$

time-varying offsets, where a=0.25 and $\delta_{0}\in {ℜ}^{nN}$ and $\delta_{1}\in {ℜ}^{nN}$ are constant vectors and have been proposed as

$$\begin{array}{cc} \delta_{0} & ={\left[\begin{array}{cccccccccccc} -1.2 & 1.4 & 2 & 1.4 & -3.6 & 0 & 3.6 & 0 & -2 & -1.4 & 1.2 & -1.4 \end{array}\right]}^{⊤}\\ \delta_{1} & ={\left[\begin{array}{cccccccccccc} 0.4 & -1.4 & 0.4 & -1.4 & -0.4 & 0 & 0.4 & 0 & -0.4 & 1.4 & -0.4 & 1.4 \end{array}\right]}^{⊤}. \end{array}$$

In this simulation, the communication graph is a directed ring and its corresponding Laplacian matrix is given by

$$L=\left[\begin{array}{cccccc} 1 & 0 & 0 & 0 & 0 & -1\\ -1 & 1 & 0 & 0 & 0 & 0\\ 0 & -1 & 1 & 0 & 0 & 0\\ 0 & 0 & -1 & 1 & 0 & 0\\ 0 & 0 & 0 & -1 & 1 & 0\\ 0 & 0 & 0 & 0 & -1 & 1 \end{array}\right].$$

To obtain a better insight into the performance of the Luenberger observer, we compute the norm of the velocity errors given

$$\begin{array}{c} \left|\left|e_{v_{x}}\right|\right|=\left|\left|{\hat{v}}_{x}-v_{x}\right|\right|,\;\left|\left|e_{v_{y}}\right|\right|=\left|\left|{\hat{v}}_{y}-v_{y}\right|\right| \end{array}$$

where

$$\begin{array}{cc} v_{x} & =\left[\begin{array}{ccc} v_{x1}^{⊤} & \cdots & v_{x6}^{⊤} \end{array}\right]\in {ℜ}^{6},\;v_{y}=\left[\begin{array}{ccc} v_{y1}^{⊤} & \cdots & v_{y6}^{⊤} \end{array}\right]\in {ℜ}^{6}\\ {\hat{v}}_{x} & =\left[\begin{array}{ccc} {\hat{v}}_{x1}^{⊤} & \cdots & {\hat{v}}_{x6}^{⊤} \end{array}\right]\in {ℜ}^{6},\;{\hat{v}}_{y}=\left[\begin{array}{ccc} {\hat{v}}_{y1}^{⊤} & \cdots & {\hat{v}}_{y6}^{⊤} \end{array}\right]\in {ℜ}^{6} \end{array}$$

**Figure 3:**
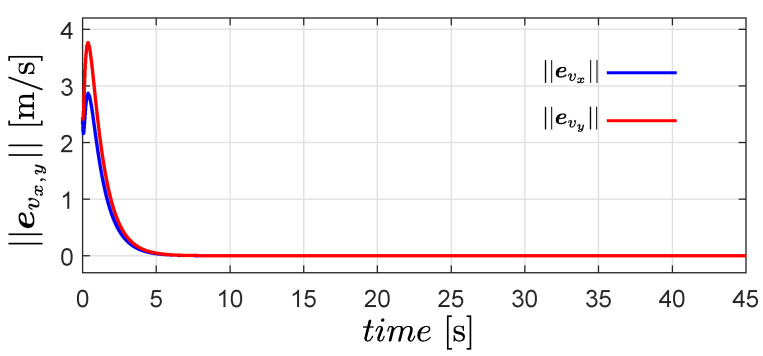
Time evolution of the quantities $\left|\left|e_{v_{x}}\right|\right|$ and $\left|\left|e_{v_{y}}\right|\right|$.

The time evolution of $\left|\left|e_{v_{x}}\right|\right|$ and $\left|\left|e_{v_{y}}\right|\right|$ is shown in Figure 3. Clearly, both quantities converge to zero exponentially; therefore, the estimated velocity converges to the actual velocity of the robots.

**Figure 4:**
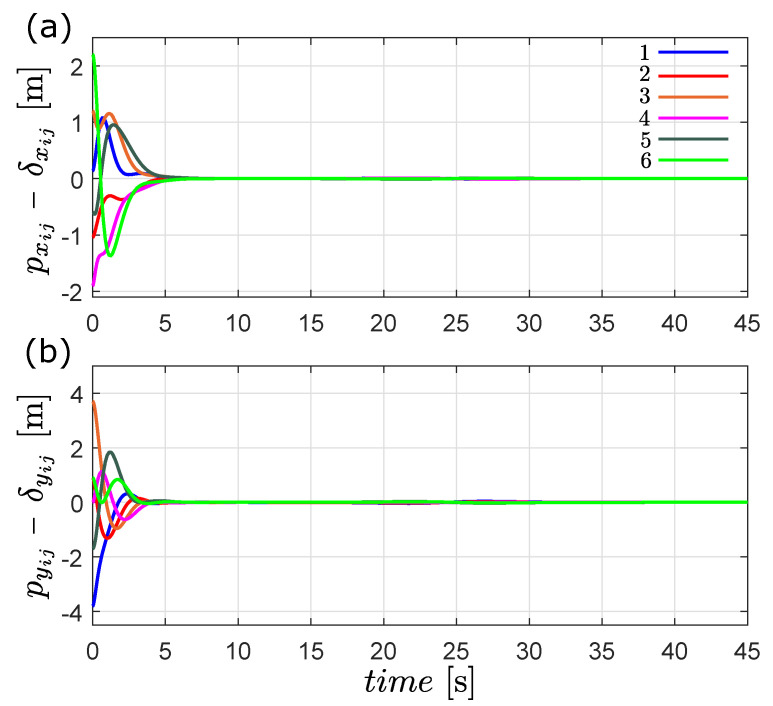
Time evolution of the formation error $p_{ij}\left(t\right)-\delta_{ij}\left(t\right)$ in the first simulation, (**a**) *x* [m] and (**b**) *y* [m].

In Figure 4, we show that the the time-varying formation is achieved, due to the fact that $p_{x_{ij}}\left(t\right)-\delta_{x_{ij}}\left(t\right)$ and $p_{y_{ij}}\left(t\right)-\delta_{y_{ij}}\left(t\right)$ converge exponentially to zero after the transient response.

**Figure 5:**
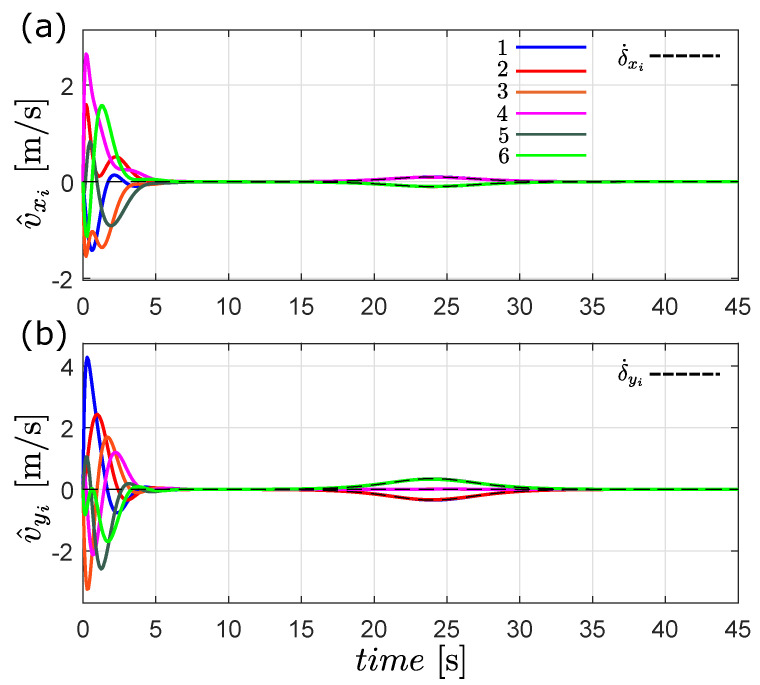
Estimated velocity of each robot, (**a**) ${\hat{v}}_{x}$ [m/s] and (**b**) ${\hat{v}}_{y}$ [m/s].

The estimated velocity is shown in Figure 5. In the figure, all robots’ estimated velocities converge to the rate of change of the formation given by ${\dot{\delta }}_{i}\left(t\right)$ after a few seconds. From the results of Figure 4 and Figure 5, we can conclude that the formation objective is achieved.

**Figure 6:**
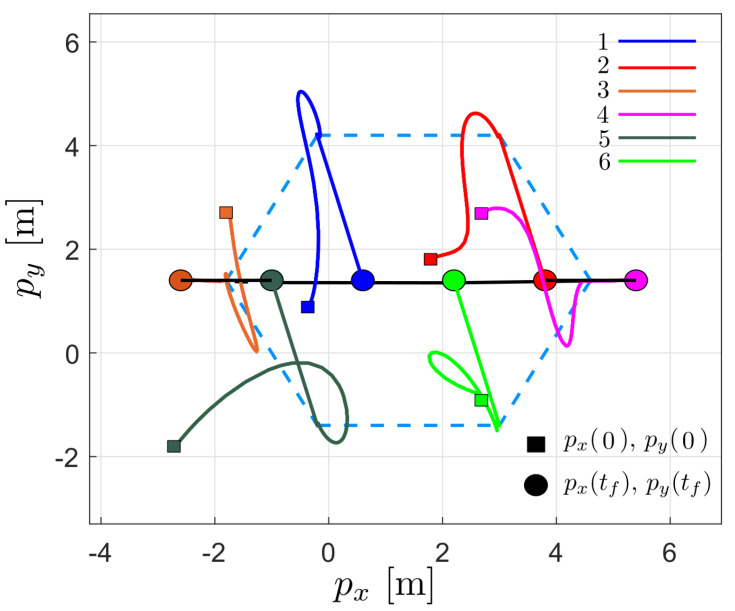
Trajectory of the robots in the plane during the first simulation; □ denotes the initial position of the robots and ◯ denotes the final position of the robots.

Figure 6 shows the robots’ trajectory in the x−y plane. As can be seen for t<6, the agents move from the initial position to achieve the formation in blue dotted lines; subsequently, for t>6, the agents move to a line formation, in which they remain for the rest of the time.

### 6.2. Flocking Control

The objective of the second simulation is to validate the performance of the flocking controller (19) together with the observer (18). The desired velocity profile is proposed as

$$v^{d}\left(t\right)=\left[\begin{array}{c} 0.6-0.1(\tanh(t-10)-\tanh(t-30))\\ -0.2(\tanh(t-10)-\tanh(t-30)) \end{array}\right].$$

It is important to point out that the first derivative of the desired velocity profile ${\dot{v}}^{d}\left(t\right)\to 0$ as t→∞; hence, the condition in Proposition 3 is fulfilled. On the other hand, the initial conditions, the control and observer gains, the time-varying formation $\delta_{i}\left(t\right)$ and the communication graph are the same as in the first simulation.

Before performing the tests to validate the flocking control, we carried out a simulation to assess the performance of the distributed observer (24). Note that the distributed observer (24) does not depend on the states of the robot network. We consider that only robots 1 and 5 know the desired velocity profile; thus, the parameter $d_{i}$ is set as $d_{1},d_{5}=1$ and $d_{2,3,4,6}=0$. This means that the rest of the robots will have to recover the information through the distributed observer. The initial condition and observer gain for the distributed observer were chosen as ${\hat{v}}_{i}^{d}\left(0\right)=0$ for all i∈N and μ=10.

**Figure 7:**
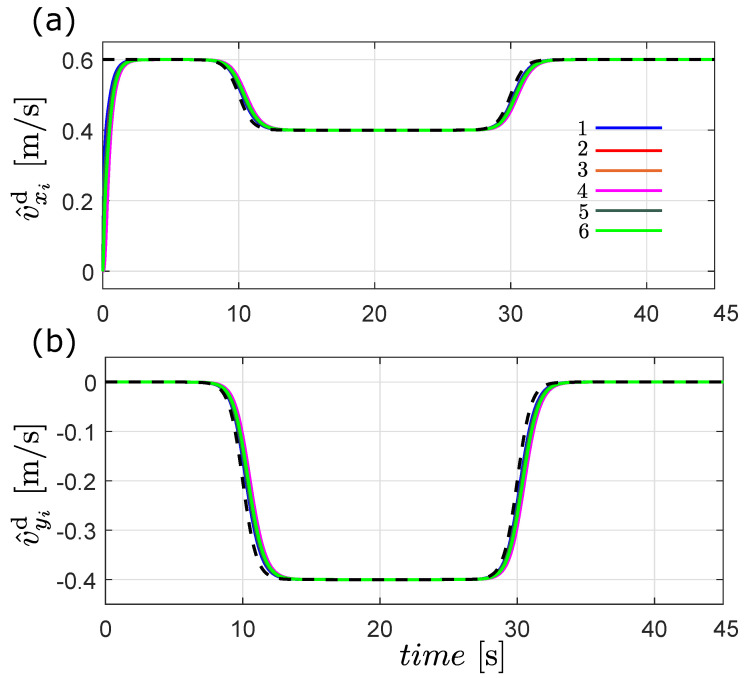
Estimated desired velocities generated by the distributed observer (24), (**a**) estimated velocity in the *x* coordinate, (**b**) estimated velocity in the *y*-component. The dotted line represents the desired velocity profile.

Figure 7 shows the trajectories obtained with the distributed observer (24). As can be observed, all the estimated states converge to the desired velocity profile in both coordinates; see Figure 7a,b. With this result, we can say that the estimator is working well; therefore, it can be used in combination with the observer-based flocking approach described in Section 4.

**Figure 8:**
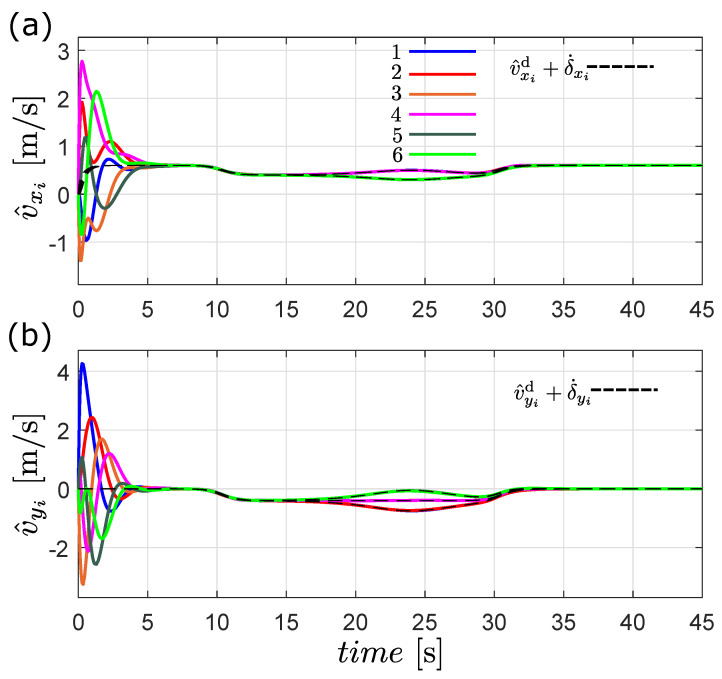
Estimated velocity of each robot in the flocking simulation, (**a**) ${\hat{v}}_{x}$ [m/s] and (**b**) ${\hat{v}}_{y}$ [m/s].

**Figure 9:**
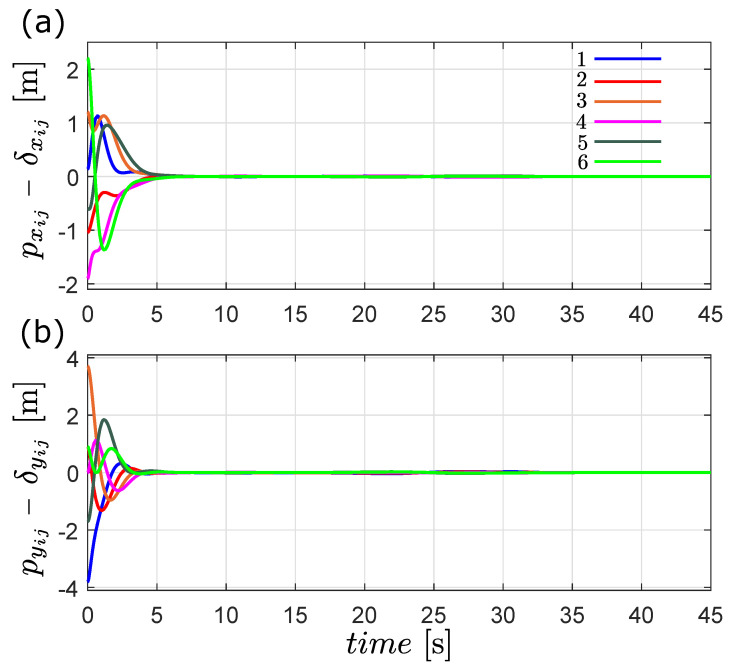
Time evolution of the distance error $p_{ij}\left(t\right)-\delta_{ij}\left(t\right)$ in the flocking simulation, (**a**) $p_{x}$ [m] and (**b**) $p_{y}$ [m].

According to the definition of flocking given in Section 2, this behavior is achieved if all the robots’ velocities follow the desired flocking velocity plus the rate of change of the formation and the robots maintain a safe distance from one another and form a desired geometric pattern. The velocity matching condition is shown in Figure 8, where all the estimated velocities of the robots converge to $v^{d}\left(t\right)+{\dot{\delta }}_{i}\left(t\right)$. The second flocking condition is depicted in Figure 9, where clearly the distance $p_{ij}\left(t\right)-\delta_{ij}\left(t\right)$ converges to zero, meaning that the robots achieve the desired formation. It is important to highlight the good performance of the observer-based flocking algorithm considering that only the position of the robots is obtained from sensor measurements and only two robots know the the desired flocking velocity profile.

**Figure 10:**
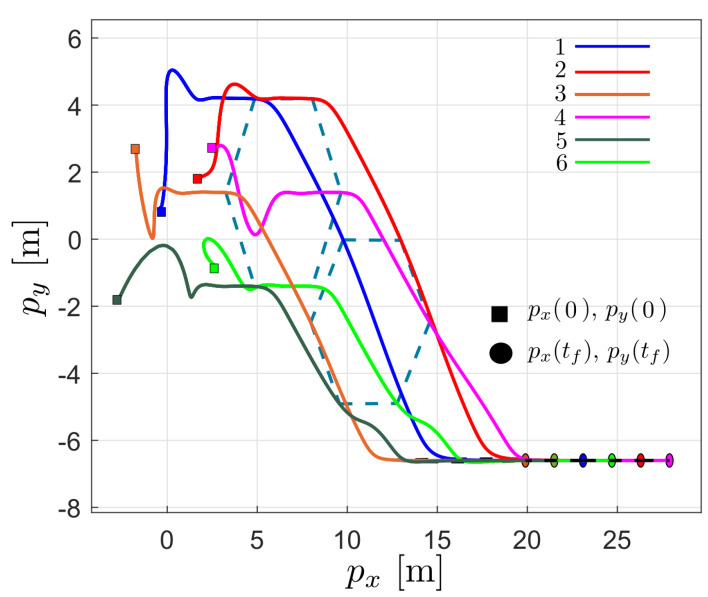
Trajectory of the robots in the plane during the flocking simulation; □ denotes the initial position of the robots and ◯ denotes the final position of the robots.

Figure 10 shows the trajectory of the robot network in the plane, which is similar to the flocking behavior observed in birds—that is, while maintaining a time-varying formation, all the robots move with the same speed. With this result and the ones presented in Figure 8 and Figure 9, we can conclude that the proposed flocking control law is able to emulate the flocking behavior.

## 7. Conclusions

In this work, we study the problem of designing control laws that achieve time-varying formation and flocking in robot networks where each robot is modeled as a double integrator system. To solve this problem, we adopt a hierarchical control strategy that allows us to design a decentralized dynamic controller able to achieve both collective behaviors. For instance, if time-varying formation is pursued, we only need to set the desired flocking velocity to zero. An important advantage of the proposed controller is that for the robot *i*, the control input $u_{i}$ does not require the linear velocities of its neighbors; it only requires the relative position of the neighbors and its own velocity.

Since, in practice, it is not always possible to measure all the states of the system, a Luenberger observer is proposed to estimate the robot’s velocity. The hierarchical control approach allows us to easily combine the formation and flocking control law with the observer, and the stability analysis is very straightforward. In addition to the velocity observer, we design a distributed observer that estimates the desired flocking velocity for those robots that do not have access to the desired velocity profile.

To model the communication between agents, the theory of graphs is employed. The proposed controller works for either undirected or directed graphs. As a result, it is possible to avoid the use of the leader–follower approach, which is most used in these types of works; the only condition is that the graph must be connected and strongly connected for the distributed observer. To validate the presented theory, numerical results are shown that allow us to conclude to that the proposed control law is able to emulate the collective behavior under study; in the case of the distributed observer, the obtained results were good, considering that only two agents knew the desired velocity profile. Finally, as future research, we will study the case of collision avoidance, so as to later implement the method presented in this work in some unmanned vehicles.
